# Unveiling the mechanisms and promising molecular targets of curcumin in pancreatic cancer through multi-dimensional data

**DOI:** 10.1038/s41598-025-05346-w

**Published:** 2025-07-01

**Authors:** HongMing Xie, JieBin Liang, HongBiao He, Zewei Zhuo, JiaXuan Li

**Affiliations:** 1 Department of Gastroenterology, Zhongshan Chenxinghai Hospital of Integrated Traditional Chinese and Western Medicine, Zhongshan, 528415 Guangdong China; 2 Department of Gastroenterology, Guangdong Provincial People’s Hospital, Guangdong Academy of Medical Sciences, Southern Medical University, Guangzhou, 510080 China

**Keywords:** Curcumin, Pancreatic cancer, Machine learning, Nomogram, Network pharmacology, Cancer microenvironment, Cancer, Biomarkers, Gastroenterology, Oncology, Risk factors

## Abstract

Pancreatic cancer (PC) is a highly aggressive and fatal malignancy, primarily affecting older males. Curcumin, a potential anti-cancer agent, has been shown to regulate key molecules in cancer progression, but its specific mechanisms in PC remain unclear. We conducted a comprehensive database search to identify curcumin-related targets in PC. Gene expression and immune correlations were analyzed using the GEO database, identifying differentially expressed hub genes (DEHGs). A method involving machine learning was employed to identify feature genes and create a nomogram, using external datasets and molecular docking for preliminary validation. Consensus clustering and subgroup comparisons were also performed based on DEHGs expression. We identified 35 DEHGs strongly associated with immune cell infiltration. Five feature genes (VIM, CTNNB1, CASP9, AREG, HIF1A) were used to build a nomogram, with the classification model showing AUC values above 0.9 in both training and validation groups. Molecular docking highlighted potential binding sites of five feature genes for curcumin. Clustering analysis categorized PC samples into four distinct subgroups: C1 and CII, which showed high expression and elevated immune cell infiltration, and C2 and CI, which exhibited the opposite pattern. Significant variations in scores of DEHG were seen between C1 and C2, in addition to between CI and CII. Curcumin may target DEHGs to influence PC, regulating immune and tumor proliferation mechanisms. These outcomes provide potential insights for medical applications and upcoming research.

**Supplementary Information:**

The online version contains supplementary material available at 10.1038/s41598-025-05346-w.

## Introduction

Pancreatic cancer (PC) is a very fatal tumor characterized by poor prognosis^1^. It ranks as the seventh leading reason for deaths connected with cancer globally and is anticipated to surpass breast cancer by 2025^2^. Characterized by early metastasis and resistance to chemotherapy and radiotherapy, radical surgical resection remains the primary treatment. However, most cases are diagnosed in progressive stages, with around 50% presenting distant metastases at diagnosis, thus missing the optimal window for surgery^3^. In spite of new advances, the 5-year survival rate is still around 7%, emphasizing the need for new biomarkers to improve early detection and management.

Curcumin, a polyphenolic substance extracted from turmeric (Curcuma longa), has been examined as a prospective therapy for PC. Historically utilized as a food ingredient and in medicinal applications^4–7^, preclinical investigations have shown curcumin’s anti-inflammatory, antioxidant, and anticancer effects by regulating multiple signaling pathways in various malignancies, comprising PC^8,9^. Curcumin’s safety and low toxicity make it attractive for therapeutic use. Clinical trials have tested curcumin’s anticancer effects, with promising results for its application in cancer treatment^10–14^. These findings suggest curcumin as a promising therapeutic for PC.

**Fig. 1:**
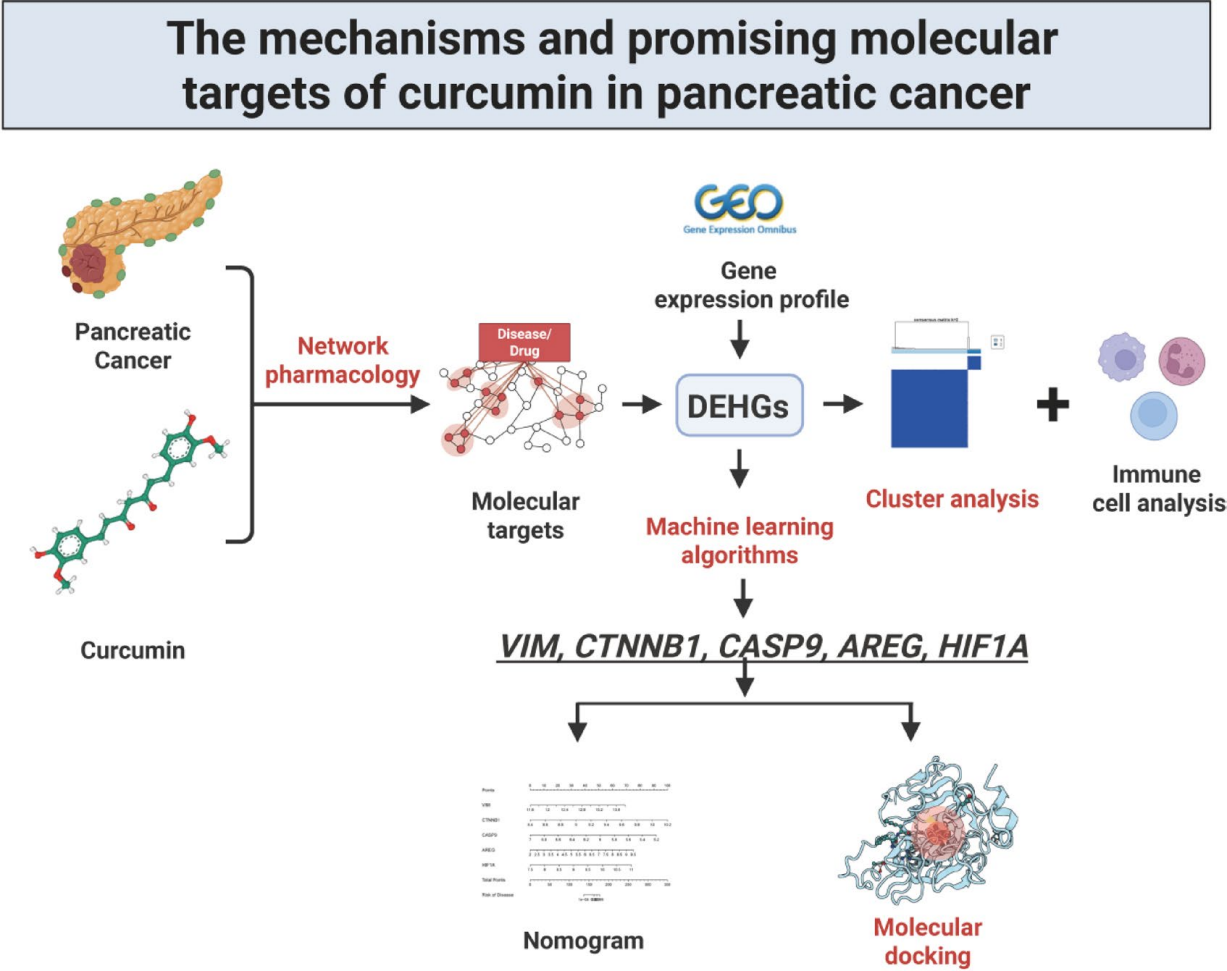
The flowchart of this study.

Current studies are limited by small sample sizes and a lack of mechanistic focus, necessitating further investigation into curcumin’s molecular mechanisms in PC. Network pharmacology, which analyzes molecular interactions between medicines and diseases from a systemic and biological network perspective, is an essential approach for identifying active components and elucidating the pathways of traditional Chinese medicine (TCM)^15^. Therefore, this investigation utilized network pharmacology to discover the core targets of curcumin in PC therapy. The primary target expression was assessed employing the dataset of Gene Expression Omnibus (GEO). Feature genes were selected using a model of machine learning, and a nomogram for PC classification was developed. Molecular docking analysis was performed to explore potential binding sites between curcumin and the core targets. Lastly, clustering analysis examined the role of differentially expressed hub genes (DEHGs) in the subgroup classification of PC, revealing curcumin’s potential mechanisms of action. The overall study procedure is illustrated in Fig. 1.

## Methods

### Components and targets in Curcumin

The structure and Isomeric SMILES of Curcumin were retrieved from the PubChem database. Target prediction was performed using SwissTargetPrediction (http://www.swisstargetprediction.ch/) and SuperPred (https://prediction.charite.de/). Additionally, target predictions were supplemented using the TCM System Pharmacology Technology Platform (TCMSP, http://tcmspw.com/tcmsp.php), HERB (http://herb.ac.cn/), and DrugBank (https://go.drugbank.com/). The predicted targets were then validated through the UniProt database. Finally, all identified targets were compiled to construct a drug-target database.

### PC targets

The investigation utilized PharmGKB (https://www.pharmgkb.org/), Online Mendelian Inheritance in Man (OMIM, https://omim.org/), and Genecards (https://www.genecards.org/) to conduct searches for “Pancreatic cancer” in order to identify targets associated with PC. The acquired targets were consolidated, and duplicates were eliminated.

### Intersection of therapeutic targets

The intersection of curcumin’s active compound targets and targets connected with PC was identified using the Venn package (Version: 1.12). These overlapping targets represent potential targets for curcumin in the intervention of PC.

### Construction of protein-protein interaction (PPI) network

The intersecting targets PPI network was constructed using the STRING database (https://string-db.org), limited to Homo sapiens and a minimum interaction score of 0.40. A minimum interaction score of 0.40 (medium confidence) was used to include a comprehensive set of potential interactions while maintaining sufficient network connectivity. For PPI network analysis, we applied the MCODE plugin of Cytoscape (version V3.8.0) with the following parameters: degree cut-off = 2, node score cut-off = 0.2, K-core = 2, and max depth = 100, to identify significant clusters within the network. Clusters with more than 10 nodes were selected, resulting in two major clusters (C1 and C2). Sub-networks were constructed for C1 and C2 based on half the average degree of nodes within each cluster. The genes within these clusters were considered key targets potentially involved in curcumin’s mechanism in pancreatic cancer.

### Data collection and processing

To obtain samples from the database of GEO (https://www.ncbi.nlm.nih.gov/geo/), we employed “Pancreatic cancer” as a keyword and limited the data kind (Expression profiling by array) and organism (Homo sapiens). Gene expression data and clinical information were obtained from the GEO database. GSE62165 (13 non-tumoral and 118 pancreatic tumor samples) was used as the training set, and GSE71729 (46 normal and 145 primary pancreatic tumor samples) served as the validation set. The raw data were preprocessed and annotated with official gene symbols, and expression levels of core genes associated with curcumin intervention in pancreatic cancer (PC) were extracted and compared between normal and PC samples.

### Expression difference of core genes, chromosome position, and expression correlation of significantly differently expressed core genes

We performed differential expression analysis of the previously identified hub genes from the PPI network using the limma package (version 3.56.2). Gene expression profiles from healthy pancreatic tissue and pancreatic cancer (PC) samples were visualized using pheatmap (version 1.0.12) and ggpubr (version 0.6.0). Hub genes with an adjusted p-value < 0.05 and |log2 fold change| ≥ 1 were defined as differentially expressed hub genes (DEHGs). The chromosomal locations of these DEHGs were plotted using the Rcircos package (version 1.2.2). In addition, the correlation coefficients among SDECGs were calculated using the “cor” function and subsequently visualized.

### Infiltration, difference, and correlation of immune cells in PC samples

The proportion of immunity cells was derived from 1,000 simulations conducted employing the CIBERSORT tool in R. The immunity cell composition in every sample was then shown employing a bar plot. The R packages “GSVA” and “GSABase” were employed to conduct Single-sample gene set enrichment analysis (ssGSEA) to evaluate the variations in immunity cell composition between the healthy and PC groups. Box plots were conducted to represent the outcomes of the ssGSEA. The scores of ssGSEA were employed to intersect the DEHGs, and the correlation coefficients were obtained employing the correlation analyses, which were then presented.

### Selection of machine learning model and nomogram for curcumin in the treatment of PC

To ensure robust and accurate identification of pancreatic cancer-related biomarkers from high-dimensional gene expression data, we employed four complementary machine learning algorithms: Generalized Linear Models (GLMs), Support Vector Machines (SVMs), Random Forests (RF), and Extreme Gradient Boosting (XGBoost). GLMs provide interpretable statistical associations by modeling both linear and nonlinear effects. SVMs are effective in handling high-dimensional data using kernel functions to focus on informative gene subsets. RF captures complex nonlinear interactions and offers feature importance rankings, while XGBoost enhances predictive performance through gradient boosting and regularization, making it suitable for sparse or imbalanced datasets. This multi-model strategy enhances the stability and reliability of feature selection. Gene expression values of differentially expressed hub genes (DEHGs) were used as input features to develop four machine learning models: GLM, SVM, RF, and XGB. The GSE62165 dataset served as the training set, while GSE71729 was used as the independent validation set. Model performance was assessed using ROC curves, AUC, reverse cumulative residual plots, and residual box plots. We compared the predictive performance of all four models, and the one with the highest AUC and lowest residual variance was selected as the optimal classifier. To identify key feature genes among DEHGs, we used the residual and ROC analyses. These feature genes and their expression profiles in normal and cancer samples were then used to construct a predictive nomogram. Decision curve analysis (DCA) and calibration plots were further applied to evaluate its clinical utility and accuracy.

### Molecular docking

The three-dimensional models of the previously acquired curcumin and feature genes were retrieved from the Protein Data Bank (PDB) (http://www.rcsb.org/) and PubChem (https://pubchem.ncbi.nlm.nih.gov). To assess the interaction of curcumin with target genes, molecular docking was performed using AutoDock Vina (version 1.2.2). The protein structures were prepared by removing water molecules and any bound ligands, followed by the addition of hydrogen atoms using AutoDockTools. Curcumin was optimized using Chem3D to minimize energy and ensure proper geometry before the docking process. Docking simulations were conducted with AutoDock Vina, using a grid box centered around the binding site of each target protein. The grid dimensions were set appropriately, with coordinates defined based on the active sites of the proteins. The most favorable binding poses were selected based on the binding energy, and the interactions were visualized using PyMOL. The binding energy for each complex was recorded. Finally, the ligand-receptor interaction patterns were analyzed using Discovery Studio Visualizer, and visual representations highlighting molecular forces such as hydrogen bonds and hydrophobic interactions were generated.

### Clusters of DEHGs and analysis between DEHGs clusters

Based on the DEHGs expression using a k-means clustering algorithm, Euclidean distance metric, and a maximum of nine clusters, the package for R called “Consensus ClusterPlus” (Version: 1.66.0) was utilized to cluster PC specimens. The resultant groups were examined by contrasting their levels of expression employing heat maps and box plots. The differentiation among clusters was evaluated by employing principal components analysis (PCA). A CIBERSORT analysis of the DEHG clusters was performed to generate a bar plot depicting the individual immunity cell composition of every specimen across the various clusters and to assess the variations in immunity cell content across the clusters. The GMT files obtained from the GSEA platform (http://www.gsea-msigdb.org/) were utilized to conduct the Kyoto Encyclopedia of Genes and Genomes (KEGG)^16–18^ and Gene ontology (GO) enrichment analyses, while the R programming language to evaluate the expression of the enrichment items across clusters was evaluated to perform Gene Set Variation Analysis (GSVA) (version 1.50.1). In the end, differential analysis was conducted on the gene expression of DEHG clusters, utilizing filtering criteria of |logFC| > 0.585 and adjusted P-Value < 0.05.

### Enrichment analysis in differentially expressed genes (DEGs) between DEHGs clusters

The DEGs within the differentially expressed hub genes (DEHGs) clusters underwent molecular function (MF), biological process (BP), and cellular component (CC) gene ontology (GO) enrichment analysis, in addition to KEGG pathway enrichment analysis. The analyses were performed employing the R software packages, including “clusterProfiler” and “enrichplot,” with a screening criterion of p-value < 0.05. The outcomes were shown as circular plots and bar graphs.

### Clusters of DEGs and analysis among DEG clusters

We performed an additional analysis of the cluster based on the DEGs expression (from GSE62165 dataset), employing the exact identical clustering algorithm as described in Sect. 2.11, and chose the DEG cluster exhibiting the best accurateness. The levels of DEGs expression across various clusters, the variations in DEHGs, and immunity cell composition across these clusters were analyzed in accordance with the DEG clustering outcomes. The data were represented employing box plots, respectively.

### DEHGs scores and differential analysis, and construction of alluvial plot

We used the PCA approach to determine the DEHGS scores for every specimen by aggregating PC1 and PC2 according to the levels of DEHGs expression (from GSE62165 dataset). Differential analysis was conducted on the DEHGS scores of DEHGs and DEG clusters employing packages in R like “limma” (version 3.56.2) and “ggpubr” (version 0.6.0). Box plots were generated to depict the DEHG scores of samples categorized into DEHGs and DEGs. An alluvial diagram was created by employing the package in R called “ggalluvial” to illustrate the linkages and mechanisms between the DEHG clusters, DEG clusters, and samples with elevated and decreased scores of DEHG.

### Statistical analysis

All statistical analyses were performed employing R version 4.3.3. This study used t-tests to compare two independent samples and the Wilcoxon paired rank sum test for two paired samples. For datasets that include three or more groups, one-way analysis of variance (ANOVA) and the Kruskal-Wallis rank sum test were utilized, while correlation analysis was conducted employing the Spearman rank correlation test. Statistical significance was determined at a P-value of less than 0.05 or a false discovery rate (FDR) adjusted P-value of less than 0.05, employing the Benjamini-Hochberg method.

## Results

### Identification of potential targets of curcumin in the management of PC

**Fig. 2:**
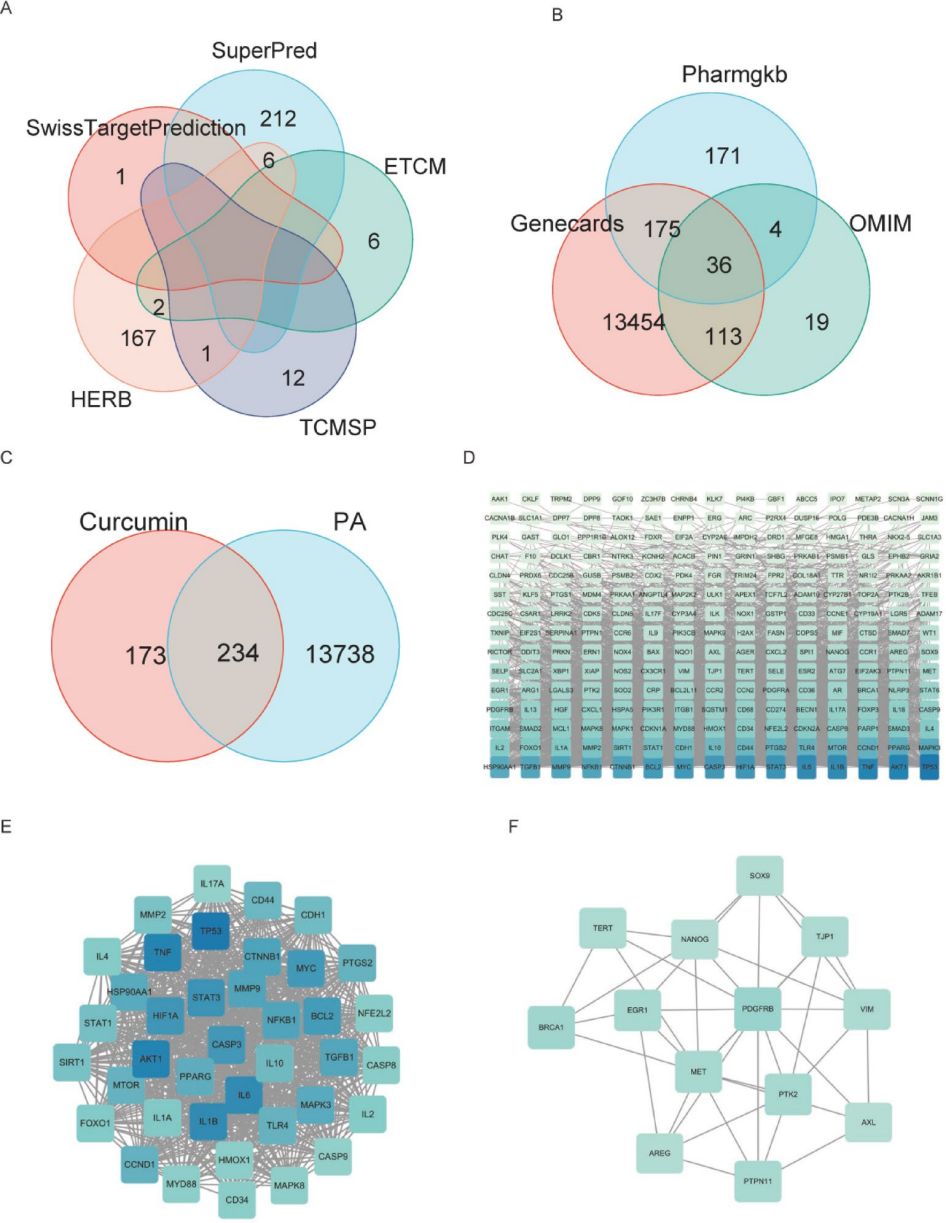
Target Analysis of Curcumin and PC. (**A**) Venn diagram of Curcumin targets obtained from five databases. (**B**) Venn diagram targets connected with PC retrieved from the GeneCards, PharmGKB, and OMIM databases. (**C**) Overlap of identified target genes between Curcumin and PC. (**D**) PPI network from STRING database visualized in Cytoscape 3.8.0. The color intensity of each node reflects its degree value: the deeper blue the color, the higher the degree; the lighter the color, the lower the degree (**E**) The C1 network cluster with more than 10 nodes identified through MCODE clustering. (**F**) The C2 network cluster with more than 10 nodes determined through MCODE clustering.

To identify the potential targets of curcumin, we searched five databases (SuperPred, SwissTargetPrediction, HERB, ETCM, and TCMSP) and identified 407 curcumin-related targets for further analysis (Fig. 2A). The PC-related targets were acquired from the databases of GeneCards, PharmGKB, and OMIM, yielding a total of 13,972 targets (Fig. 2B). By intersecting drug and disease targets through a Venn diagram, 234 overlapping targets were found, indicating the potential therapeutic effects of curcumin against PC (Fig. 2C). Figure 2D illustrates the PPI network of these targets. To identify key targets, we utilized the MCODE plugin for clustering the PPI network, which resulted in two clusters, C1 and C2, each containing more than 10 nodes (Fig. 2E and F). Ultimately, we identified 52 key targets, considered the primary targets of curcumin in PC, including MYC, MAPK8, IL6, CDH1, CCND1, VIM, CTNNB1, CASP9, AREG, and HIF1 A. For more comprehensive information on the key targets, please refer to Supplementary Table 1.

### DEHG expression differences, chromosomal localization, and intergene associations between PC and normal tissues

**Fig. 3:**
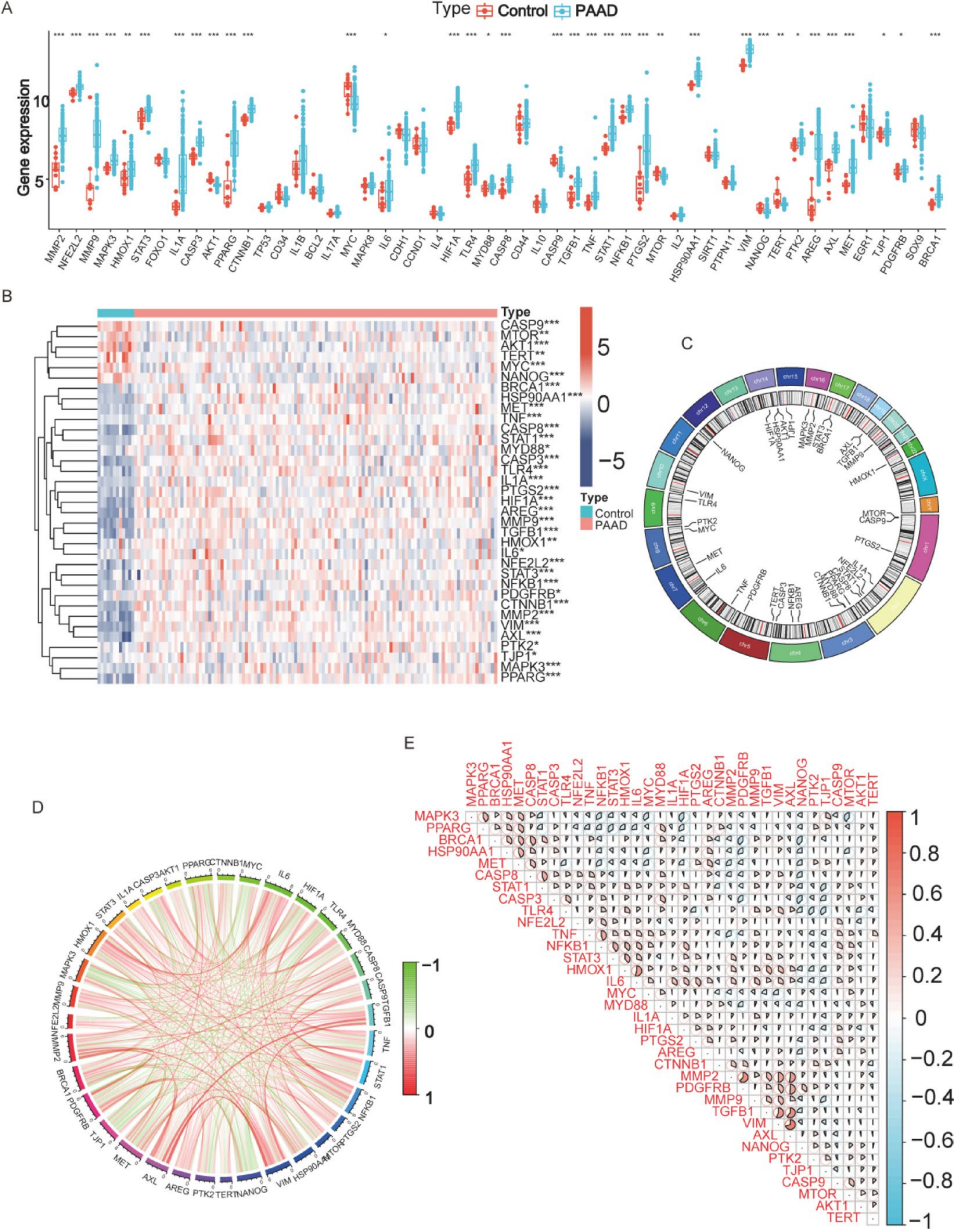
Expression and Correlation Heatmap of Key Hub Genes. (**A**) Boxplot displaying the differential key genes expression between control and PC tissues. Red represents control samples, and blue represents PC tissues. ***p < 0.001, **p < 0.01, *p < 0.05. (**B**) Heatmap depicting the expression profiles of significantly differentially expressed genes in control vs. PC specimens. The color gradient from red to blue represents high to low expression levels. (**C**) Chromosomal distribution of the differentially expressed genes (DEGs). Each bar indicates the number and position of DEGs mapped to specific chromosomal regions, providing a genomic overview of their spatial organization. (**D**) Chord diagram displaying pairwise correlations among selected DEGs. The color of the connecting ribbons represents the direction and strength of correlation: red indicates strong positive correlation (approaching + 1), and green indicates strong negative correlation (approaching − 1). (**E**) Gene-gene correlation heatmap, with red representing strong positive correlations and blue showing negative associations.

The boxplot (Fig. 3A) displays the levels of key hub genes expression in PC tissues compared to control tissues. The heatmap (Fig. 3B) highlights the 35 DEHBs between control and PC samples. Among them, CASP9, MTOR, AKT1, TERT, MYC, and NANOG are highly expressed in the normal group, while BRCA1, HSP90 AA1, MET, TNF, CASP8, STAT1, MYD88, CASP3, TLR4, IL1 A, PTGS2, HIF1 A, AREG, MMP9, TGFB1, HMOX1, IL6, NFE2L2, STAT3, NFKB1, PDGFRB, CTNNB1, MMP2, VIM, AXL, PTK2, TJP1, MAPK3, and PPARG are highly expressed in the PC group. Figure 3C displays the chromosomal locations of these genes. Both the chord diagram and the correlation heatmap demonstrate strong relationships between these genes (Fig. 3D-E).

### Correlation of DEHBs with immune cells in PC

**Fig. 4:**
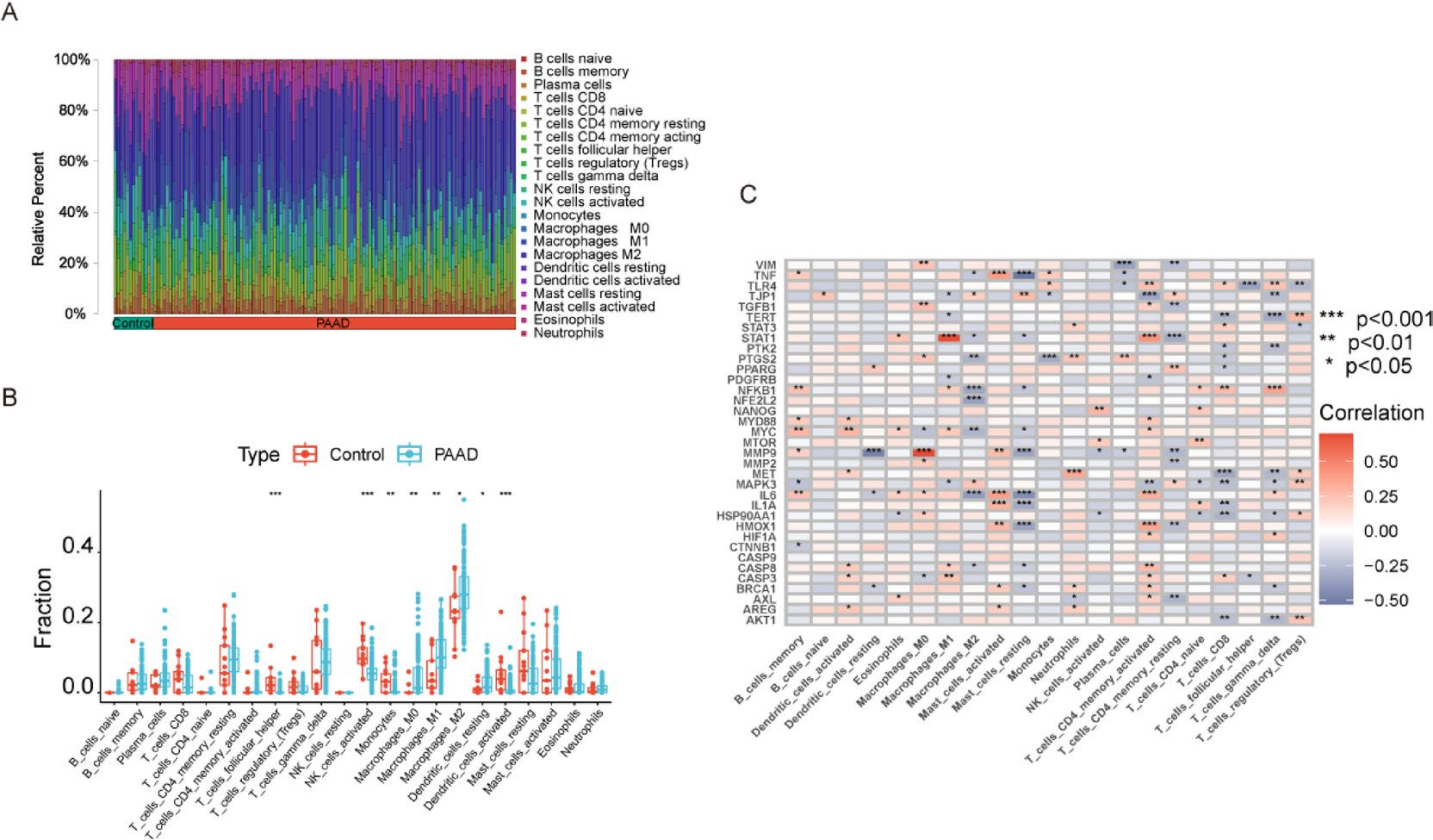
Correlation of Immune Cell Infiltration and DEHBs. (**A**) Bar plot showing the relative abundance of immune cell kinds in control and PC tissues. The x-axis represents the sample groups (control vs. PC), and the y-axis shows the relative percentage of each immune cell type, with different colors indicating various immune cell populations. (**B**) Box plot showing the proportions of immunity cell types in control and PC tissues. Red represents control samples, and blue represents PC samples. **p < 0.001, **p < 0.01, *p < 0.05. (**C**) Heatmap illustrating the correlations between immune cell types and DEHBs. The color gradient indicates correlation values (red for positive correlation, blue for negative correlation). ***p < 0.001, **p < 0.01, *p < 0.05.

To discover the pathways of DEHBs in the PC group from various perspectives, we conducted an immunity cell infiltration analysis. First, we analyzed the relative abundance of various immunity cell kinds in PC and control samples (Fig. 4A). Figure 4B presents a box plot of immune cell proportions, revealing that activated dendritic cells, regulatory T cells (Tregs), and M2 macrophages were significantly more abundant in PC samples compared to controls. In contrast, resting mast and naive B cells were more prevalent in control tissues. These findings suggest that PC is associated with an immunosuppressive environment, potentially contributing to tumor progression. Figure 4C displays a heatmap demonstrating the connection between immune cell types and key hub genes. Notably, genes such as IL6 and NFKB1 were strongly positively correlated with immune-activating cells (e.g., activated mast and memory CD4 + T cells), while genes like MAPK3, MET, and TERT showed significant negative correlations with cells suppressed by immunity, like M2 macrophages and Tregs. These correlations underscore the possible function of these genes in modulating the immune microenvironment in PC and suggest their value as therapeutic targets.

### Development of PC prediction models depending on DEHGs

**Fig. 5:**
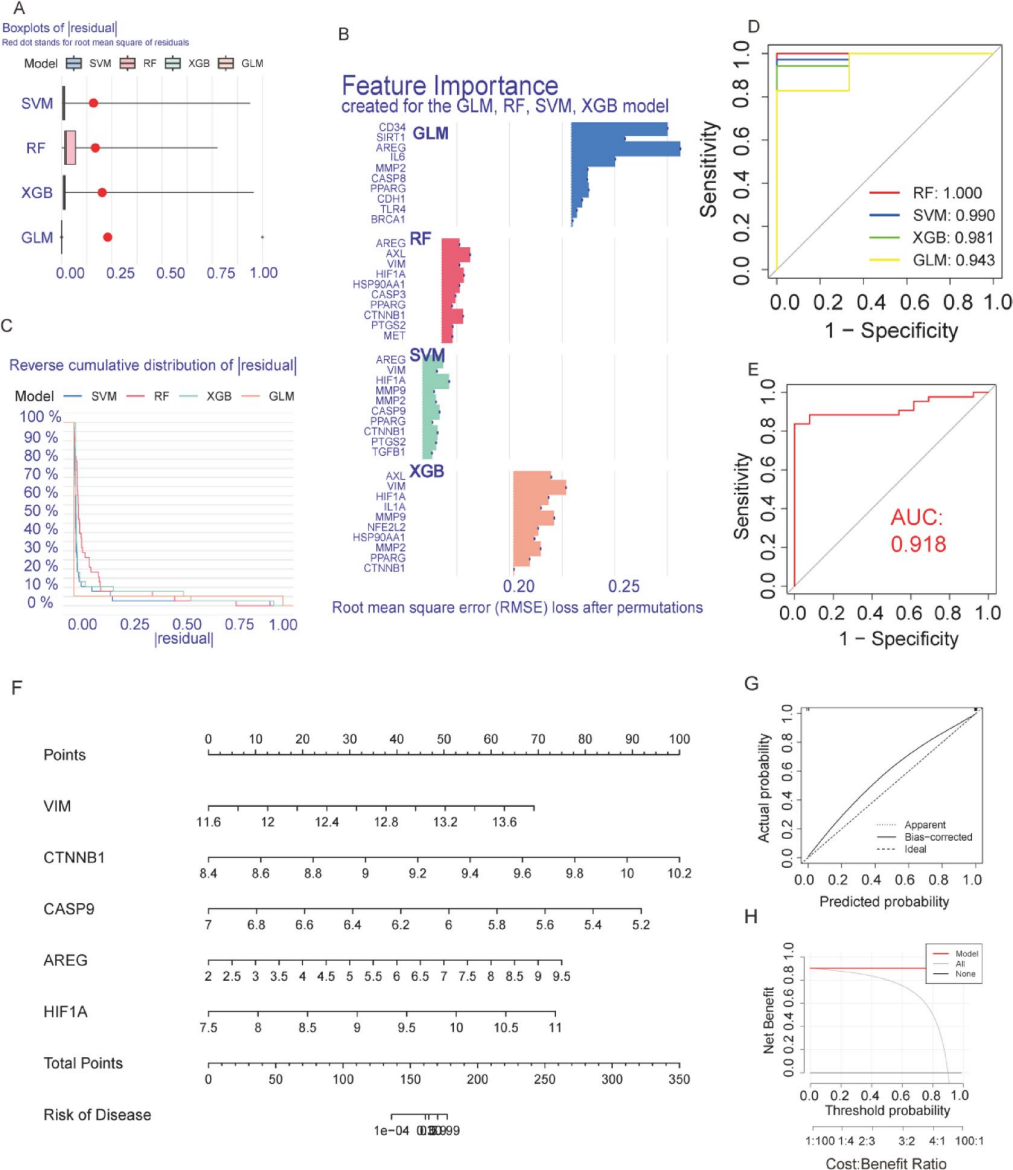
Performance and Feature Importance of PC Prediction Models. (**A**) Boxplots of residuals for RF, SVM, XGB, and GLM models in the training cohorts, with lower residuals indicating better performance. (**B**) Feature importance analysis of the models in the training cohort (GSE62165), highlighting the key genes contributing to predictive accuracy. (**C**) Cumulative distribution of residuals for each model, with steeper curves indicating better performance. (**D**) ROC curves for the four models in the training cohort. (**E**) ROC curves in the validation cohort for SVM models. (**F**) Nomogram predicting disease risk based on molecular markers VIM, CTNNB1, CASP9, AREG, and HIF1 A. Each marker contributes a corresponding score, and the total score correlates with overall disease risk. (**G**) Calibration curve comparing predicted probabilities to actual outcomes. (**H**) Decision curve analysis assessing the medical utility of the nomogram model, showing higher net benefit across threshold probabilities compared to no model.

We constructed various machine learning prediction models based on DEHG expression data. In the training cohort (GSE62165), Fig. 5A displays the boxplots of residuals for four models: RF, SVM, XGB, and GLM. The RF and SVM models demonstrated the lowest residuals and the steepest cumulative distribution of residuals (Fig. 5C), indicating superior predictive performance. Figure 5B shows the feature importance of each model, highlighting the genes that contributed most to predictive accuracy. The ROC curves for all models are shown in Fig. 5D, with the RF model achieving the uppermost AUC of 1.000, followed by SVM (0.980), XGB (0.981), and GLM (0.943), confirming that RF and SVM had the best sensitivity and specificity in the training set. In the validation cohort (GSE71729), Supplementary Figs. 1 A-B again display the boxplots and cumulative distribution of residuals for each model. In the validation cohort, SVM achieved the highest AUC of 0.918 (Fig. 5E), followed by RF (0.897), XGB (0.884), and GLM (0.687) (Supplementary Fig. 1 C-E). These results further validate the robustness of the SVM models in predicting PC. In the SVM model, we selected the top five genes with the maximum importance scores (VIM, CTNNB1, CASP9, AREG, and HIF1 A) to construct a nomogram (Fig. 5F). Patients can predict the probability of developing PC by calculating the total score depending on the expression levels of these key genes. Figure 5G shows the calibration curve of the nomogram, indicating consistency between anticipated and actual probabilities. The close alignment of the curves suggests good predictive accuracy. Figure 5H presents the decision curve analysis, where the red line represents the model, demonstrating net benefit across a range of threshold probabilities. The model consistently outperforms the “no model” strategy, highlighting its clinical utility.

### Molecular docking analysis based on five molecular markers

**Fig. 6:**
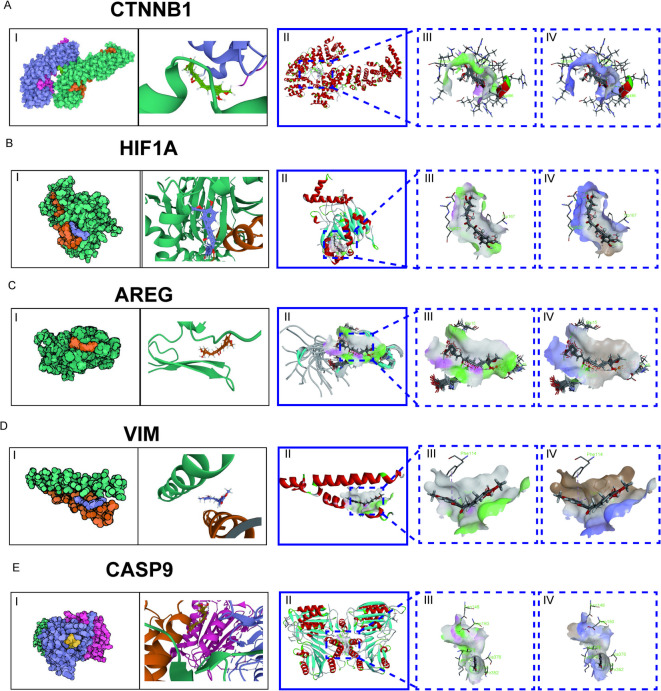
The molecular docking models of curcumin with the five molecular markers. (**A**) CTNNB1. (**B**) HIF1 A. (**C**) AREG. (**D**) VIM. (**E**) CASP9. (**I**) Cartoon representations showing the superimposed structures of curcumin and the corresponding protein targets. (**II**) Three-dimensional visualization of the binding pockets generated using Discovery Studio. Non-bonded interactions between receptor and ligand atoms are depicted as dashed lines in various colors. Bold lines highlight the ligand and receptor residues directly involved in binding, while lighter lines indicate surrounding residues forming the pocket. (**III**) Hydrogen bond interaction models: receptor residues acting as hydrogen bond donors are shown beneath pink surfaces, while acceptors are shown beneath cyan surfaces. (**IV**) Hydrophobic interaction models: the color gradient ranges from brown (indicating highly hydrophobic regions) to blue (indicating less hydrophobic regions). Green labels indicate amino acid three-letter codes and corresponding residue IDs.

To investigate the potential binding between curcumin and the proteins encoded by the five key molecular markers identified in our analysis (CTNNB1, HIF1 A, AREG, VIM, and CASP9), we performed molecular docking using AutoDock Vina v1.2.2. The 3D structure of curcumin was retrieved from the PubChem database (CID: 969516), and the crystal or NMR structures of the five protein targets were obtained from the RCSB PDB database: CTNNB1 (PDB ID: 1 g3j), HIF1 A (1 h2k), AREG (2rnl), VIM (3 g1e), and CASP9 (1jxq). Prior to docking, all structures were preprocessed by removing water molecules, adding polar hydrogens, and converting to PDBQT format. Grid boxes were centered around the functional domains of each protein to ensure adequate coverage of potential binding pockets. Docking results revealed that curcumin formed stable complexes with all five proteins, with binding energies below − 5.0 kcal/mol, indicating strong and favorable interactions (Supplementary Table 2). The docking poses demonstrated that curcumin occupied hydrophobic pockets and established hydrogen bonds and electrostatic interactions with key residues at the binding sites (Fig. 6A-E).

### Identification of two PC subtypes based on DEHGs

**Fig. 7:**
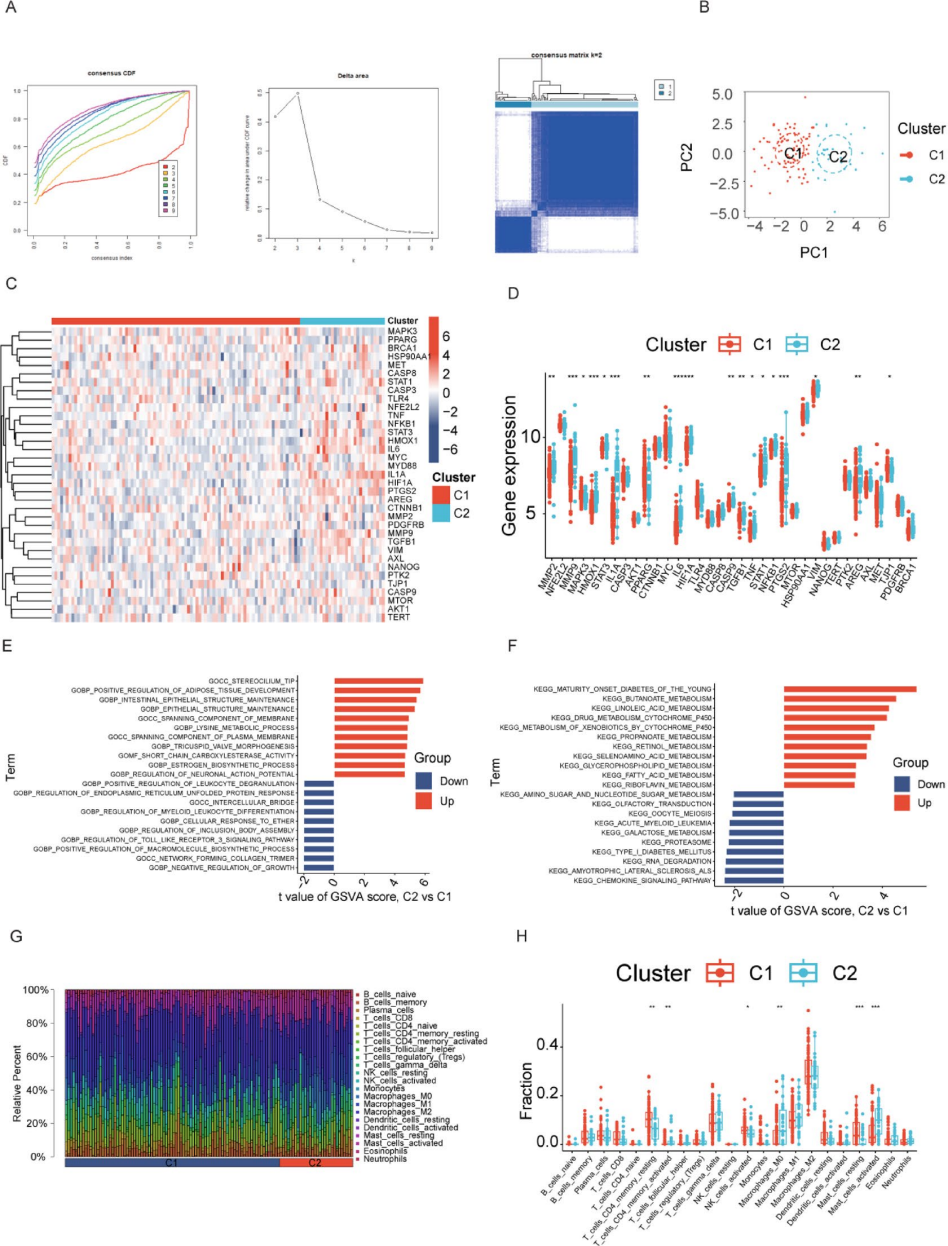
Analysis of Two Molecular Subtypes of PC Samples. (**A**) Consensus cumulative distribution function (CDF) curves and delta area plot, with a consensus heatmap identifying two distinct subtypes (C1 and C2). (**B**) PCA plot demonstrating sample distribution across the two subtypes. (**C**) Heatmap of differential gene expression, where red shows high expression and blue shows low expression. (**D**) Boxplots showing the expression of DEHGs in the two subtypes, with red for C1 and blue for C2. (E, F) GSVA results display the GO biological processes (**E**) and KEGG pathways (**F**) enriched in each subtype. Red bars represent processes enriched in C2, while blue bars represent those enriched in C1. (**G**) Immune cell infiltration landscape for the two subtypes. (**H**) Boxplots illustrating the proportion of different immunity cell types between C1 and C2.

Using unsupervised K-means consensus clustering, two distinct subtypes of PC were identified based on 35 DEHGs. The optimal clustering was determined when K = 2 (Fig. 7A). PCA further confirmed a clear distinction between the two clusters (Fig. 7B). In contrast to Cluster 1, 15 DEHGs, except for MAPK3, PPARG, and TJP1, were significantly upregulated in Cluster 2 (Fig. 7C-D). To explore the biological processes and pathways involved in each subtype, we performed GSVA. GO analysis showed that processes related to cell proliferation and immune evasion, such as Positive regulation of macromolecule biosynthetic process, Epithelial structure maintenance, and Regulation of myeloid leukocyte differentiation, were upregulated in Cluster 2 (Fig. 7E). On the other hand, functions related to cell inhibition, such as Negative modulation of growth, were enriched in Cluster 1. Analysis of the KEGG pathway revealed that mechanisms connected to metabolism and proliferation, like Insulin resistance, Oxidative phosphorylation, and Cell cycle, were activated in Cluster 2 (Fig. 7F), while immune regulation and cellular homeostasis pathways, including the NOD-like receptor signaling pathway, Autophagy, and Phosphatidylinositol signaling system, were activated in Cluster 1. Finally, Fig. 7G illustrates the immune cell infiltration in the two subtypes. Cluster 1 exhibited higher levels of immune-effector cells, such as resting memory CD4 + T cells and activated NK cells. The box plot in Fig. 7H further quantified the significant variations in the proportions of various immunity cell types between the two groups.

### Comprehensive analysis of the molecular characteristics and immune microenvironment of DEHGs subtypes

**Fig. 8:**
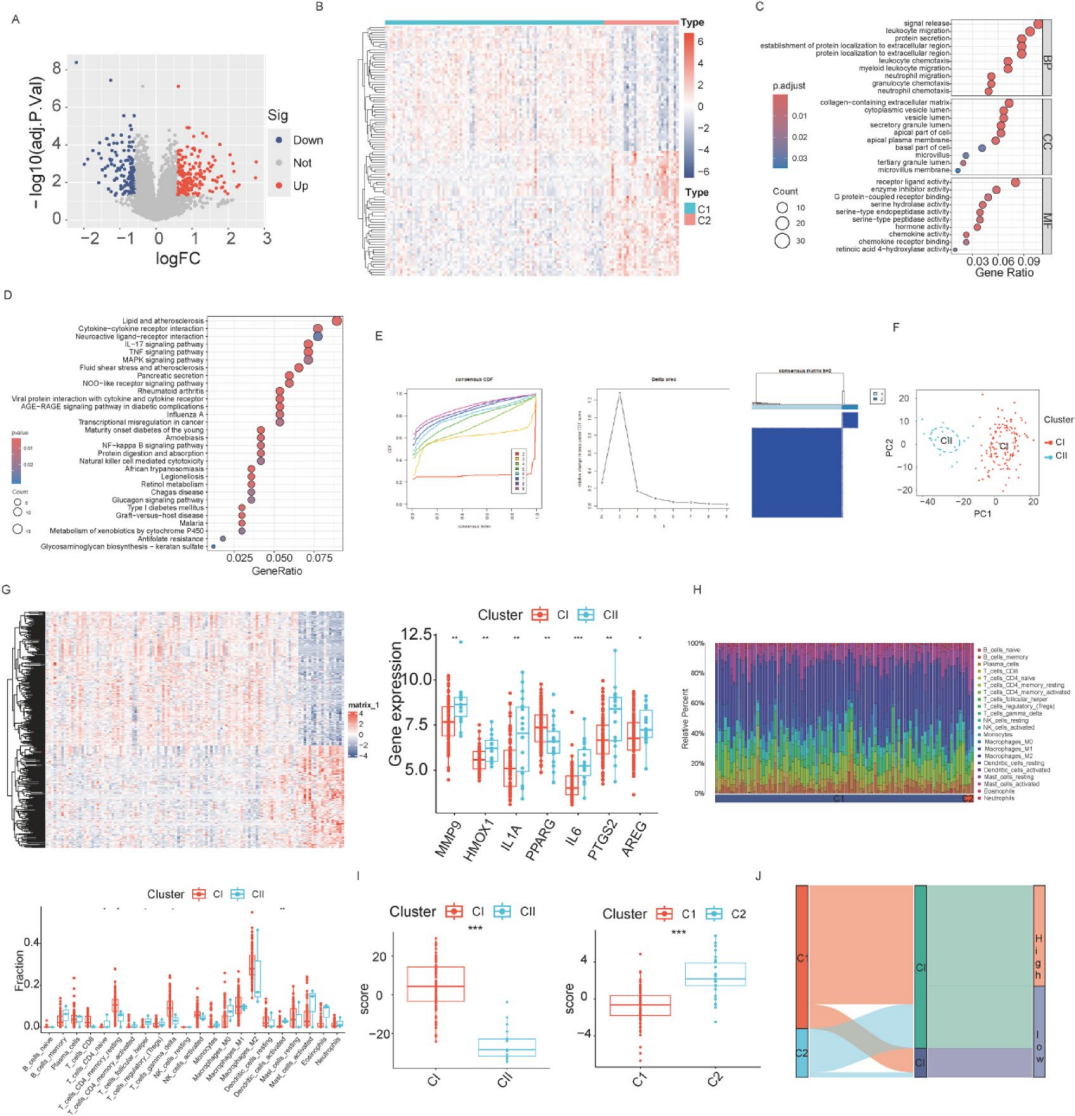
Analysis of Molecular Characteristics and Immune Microenvironment of DEHGs Subtypes. (**A**) Volcano plot displaying differentially expressed genes between the two subtypes, with red representing upregulated genes and blue representing downregulated genes. (**B**) Heatmap of gene expression, demonstrating clustering of samples into two distinct subtypes. (**C**) GO enrichment analysis of biological processes involved in each subtype. (**D**) Top 20 enriched pathways between the subtypes, showing significant involvement of immune and metabolic pathways. (**E**) Consensus clustering CDF curve and delta area plot, identifying the optimal number of clusters (K = 2). (**F**) PCA plot displaying the separation between the two subtypes. (**G**) Expression patterns of DEGs and DEHGs in clusters CI and CII. (**H**) Box plot showing differential expression analysis of DEHG scores between DEG clusters. (**I**) Box plot displaying differential expression analysis of DEHG scores between DEHG clusters. (J) Alluvial diagram illustrating the correspondence between different sample clusters.

To better understand the gene expression patterns between the subtypes and explore the underlying biological mechanisms, we screened for DEGs between clusters C1 and C2. In the volcano plot (Fig. 8A), the DEGs are highlighted, showing significantly upregulated (red) and downregulated (blue) genes between the two subtypes. The heatmap analysis (Fig. 8B) illustrates the clustering of these DEGs across samples, revealing clear expression differences between the two clusters (C1 and C2), suggesting distinct molecular signatures. Figure 8C presents the GO enrichment analysis, which highlights key biological processes associated with each subtype. Enrichment of pathways related to signal transduction, protein localization, and regulation of cell morphogenesis in one subtype suggests a more aggressive tumor behavior, whereas the other subtype shows enrichment in immune response and metabolic processes. Figure 8D presents the top 20 pathways significantly enriched in both subtypes, with pathways like cytokine-cytokine receptor interaction, Toll-like receptor signaling, and immune-related pathways being particularly prominent in Cluster 2, indicating that Cluster 2 may have a more active immune microenvironment. Figure 8E shows the consensus clustering analysis, identifying the optimal number of clusters (K = 2) depending on the CDF curve and delta area plot. Figure 8F further supports this division with PCA, demonstrating a clear separation between the two subtypes (CI and CII). Figure 8G presents the heatmap of differential gene expression in CI and CII, as well as the box plots of DEHGs in CI and CII. Results show that DEHGs such as MMP9, HMOX1, IL1 A, IL6, PTGS2, and AREG are upregulated in CII, while PPARG is downregulated in CII. Figure 8H displays the immune infiltration landscape, where Cluster II is associated with a higher proportion of immunosuppressive cells, like M2 macrophages and regulatory T cells, suggesting that this subtype might be more immune evasive. Comparing the score differences between clusters, we evaluated whether there were significant differences in DEHGs between clusters to confirm the robustness of the outcomes. PCA-based analysis of DEHGs clustering variations indicated a statistically significant disparity between the two clusters (Fig. 8I), with Cluster 2 exhibiting a greater score and Cluster 1 a lower score. Likewise, a statistically significant disparity in DEG values was seen between the two groups, with CI achieving higher scores and CII attaining lower levels. Figure 8J illustrates that DEHGs cluster C1 mostly aligns with DEG cluster CII, while DEHGs cluster C2 correlates to DEG cluster CI. Furthermore, elevated and diminished DEHG scores mostly align with DEG clusters CI and CII, respectively.

## Discussion

Pancreatic cancer is a highly aggressive disease that is often diagnosed at a progressive stage, leaving limited treatment options. In recent years, research has indicated that curcumin, a natural compound, may have a potential role in the management of PC^19^. Nevertheless, the particular pathways by which curcumin targets the disease are still unclear. In this investigation, we utilized network pharmacology, molecular docking, and machine learning models to identify DEHGs associated with PC. By analyzing the interactions between curcumin and pathogenic targets of PC, we aimed to deepen our understanding of curcumin’s multi-target characteristics, providing a theoretical foundation for its application in PC treatment. This approach not only helps elucidate the mechanisms of curcumin’s action but may also offer new insights for developing innovative therapeutic strategies.

Our research yielded several important findings. First, through a “drug-disease-target” network analysis, we discovered that curcumin’s therapeutic effects on PC are primarily mediated through multiple molecular targets. We utilized a PPI network to identify core targets, followed by differential expression analysis of these targets in the GEO dataset to recognize DEHGs. Machine learning further refined the selection of these genes, highlighting five key feature genes (VIM, CTNNB1, CASP9, AREG, and HIF1 A) as significant targets in PC. These genes have vital roles in the occurrence and progression of the disease, potentially influencing the biological characteristics of tumors and patient prognosis. For instance, VIM is essential in the epithelial-mesenchymal transition (EMT) process in PC cells^20^. The CTNNB1 gene, which encodes β-catenin, often shows abnormal activation in PC cells, closely linked to tumor proliferation, survival, and metastasis^21^. CASP9 is closely related to the apoptosis process, with studies showing that it is frequently downregulated in PC cells, leading to chemotherapy resistance and promoting tumor survival. AREG also contributes to tumor progression by mediating the EMT process. Its expression in PC cell lines can significantly impact cell migration and invasion capabilities^22^. Additionally, under hypoxic conditions, HIF1 A binds to the promoter of the fasciculin protein, resulting in increased levels of this protein, which is connected with poor differentiation and prognosis in PC^23^. These results underscore the function of these genes in promoting the invasive characteristics of PC cells. A comprehensive knowledge of these genes and their interactions is vital for developing new therapeutic strategies.

Furthermore, we created a nomogram utilizing the five feature genes determined by SVM to quantitatively evaluate the incidence of PC and evaluate the curcumin treatment sensitivity and accuracy. Molecular docking and analysis of the dataset of GEO were utilized to validate the interactions of these feature genes and the model construction. Our study demonstrates that the constructed nomogram effectively distinguishes between normal and PC groups, showing good clinical utility for risk prediction in PC. While CT and MRI are crucial for staging pancreatic cancer, they often miss early or subtle cases^24^. Our gene-based nomogram, built on the expression of VIM, CTNNB1, CASP9, AREG, and HIF1 A, offers a simplified yet effective risk assessment tool. Decision curve analysis shows consistent net benefit across thresholds, highlighting its potential to aid early risk stratification and guide clinical decisions. This model complements traditional imaging by providing an interpretable, molecular-based approach to patient evaluation.

The molecular docking of curcumin with PC cells not only elucidates its binding affinity but also provides insights into its mechanisms of action. In our study, docking analysis revealed that curcumin stably binds to the five key targets, with binding energies all below − 5.0 kcal/mol, indicating favorable and stable interactions. The exceptionally low binding energy observed with AREG (–147.879 kcal/mol), despite being an outlier possibly due to structural or computational factors, suggests particularly strong binding. The other targets also demonstrated significant binding affinities (CASP9: − 11.496 kcal/mol; HIF1 A: − 9.771 kcal/mol; CTNNB1: − 9.705 kcal/mol; VIM: − 8.371 kcal/mol). Functionally, these targets regulate key pathogenic pathways such as EMT, apoptosis, and hypoxia response, which are critical for PC progression. The combination of computational binding stability and known biological roles supports the hypothesis that curcumin may exert therapeutic effects by modulating these proteins^20–23,25^. While molecular docking provides a theoretical basis, further experimental validation will be necessary to confirm these interactions and their therapeutic relevance. Nevertheless, this integrated approach offers a solid foundation for understanding the multi-targeted mechanisms of curcumin in PC treatment.

The two molecular subtypes of PC identified based on DEHGs exhibit distinct transcriptional landscapes that profoundly influence tumor progression, immune evasion, and microenvironmental remodeling. To better understand the biological underpinnings of this stratification, we examined the interplay among the differentially expressed genes and their associated pathways. Cluster 2 displayed significant upregulation of genes such as MMP2, HMOX1, STAT3, IL1 A, IL6, HIF1 A, CASP9, TGFB1, TNF, STAT1, NFKB1, HSP90 AA1, PTGS2, and AREG. These genes are widely recognized for promoting tumor proliferation, inflammation, apoptosis resistance, and immune evasion. For instance, STAT3, IL6, and TNF are key inflammatory mediators that activate downstream NF-κB signaling and support a tumor-permissive microenvironment^26,27^. MMP2 facilitates extracellular matrix degradation and invasion^28^, while HIF1 A and HMOX1 contribute to hypoxia tolerance and oxidative stress response^29^, respectively. The activation of TGFB1 and PTGS2 indicates involvement in epithelial–mesenchymal transition (EMT) and immunosuppressive signaling^30,31^. On the other hand, Cluster 1 was marked by the upregulation of MAPK3, PPARG, and TJP1, genes known for their roles in maintaining epithelial integrity, modulating cell growth, and promoting anti-inflammatory responses. PPARG acts as a transcriptional regulator of lipid metabolism and is linked to immune homeostasis^32^. TJP1 (tight junction protein 1) is critical for preserving epithelial barrier function^33^, while MAPK3 is involved in mitogen-activated signaling and may counterbalance inflammatory signaling^34^. These gene expression patterns are aligned with our GSVA results, where Cluster 2 showed enrichment in proliferative and metabolic pathways, including oxidative phosphorylation and insulin resistance, whereas Cluster 1 was enriched in pathways related to immune surveillance, autophagy, and cellular homeostasis, such as the NOD-like receptor signaling and phosphatidylinositol signaling system. Therefore, Cluster 2 represents a more aggressive and immune-suppressive molecular subtype of PC, while Cluster 1 maintains epithelial characteristics and a more active immune microenvironment. This stratification has potential implications for prognosis and therapeutic targeting.

Lastly, we explored the potential pathways of curcumin’s effects on PC from an immune perspective. ssGSEA analysis exhibited statistically significant variations in the expression of certain immune cells between the normal and PC groups. Correlation analysis of DEHG expression with immune cells indicated that genes such as IL6 and NFKB1 were strongly positively correlated with immune-activating cells (e.g., activated mast and memory CD4 + T cells)^35,36^, while genes like MAPK3, MET, and TERT showed significant negative correlations with immunosuppressive cells, like regulatory T cells (Tregs) and M2 macrophages^37–39^. These correlations underscore the potential roles of these genes in regulating the immune microenvironment in PC and highlight their value as therapeutic targets. Clustering analysis further extended these findings, suggesting that the mechanisms through which curcumin treats PC are related to immune pathways such as IL17, TNF, NF-kB, and NOD-like receptors. These results align with the current understanding that curcumin’s therapeutic mechanisms primarily involve modulating inflammatory responses, blocking pro-cancer signaling pathways, and improving the immune microenvironment^40–44^, thereby suppressing the proliferation and metastasis of PC cells through various means.

Overall, our analysis through molecular docking and network pharmacology validates the multi-target mechanisms of curcumin in PC, providing a theoretical basis for its potential use as an adjuvant treatment. However, the current study is primarily based on network pharmacology and bioinformatics analyses; the efficacy of curcumin in experimental inflammation and clinical applications still requires further verification. Due to the high computational demands and resource constraints, molecular dynamics simulations were not performed at this stage. We acknowledge this as a limitation and plan to incorporate MD analysis in future work to further validate the dynamic stability of the complexes. Future research should incorporate large-scale clinical trials to explore the effects of curcumin in combination with other treatment modalities, such as radiotherapy and chemotherapy, to offer more therapeutic choices for individuals with PC.

## Conclusion

This study integrates multiple databases and machine learning models to reveal the key targets and regulatory mechanisms of curcumin in PC, particularly in relation to immune cell infiltration. We identified 35 DEHGs, among which five feature genes (VIM, CTNNB1, CASP9, AREG, HIF1 A) were utilized to construct a nomogram model with clinical predictive value. Molecular docking results suggest that these genes may serve as potential binding sites for curcumin. Furthermore, clustering analysis based on DEHGs expression categorized PC samples into four subgroups, revealing significant differences in immune infiltration and gene expression among them. These findings provide new directions for future clinical research, indicating that curcumin may serve as a potential adjuvant drug in PC immunotherapy.

## Electronic supplementary material

Below is the link to the electronic supplementary material.

Supplementary Material 1

## Data Availability

All data produced or examined during this investigation are included in this publication.
