# Supplementary material for: Unveiling the mechanisms and promising molecular targets of curcumin in pancreatic cancer through multi-dimensional data
Source: Sci Rep. 2025 Jul 1;15:21951. doi: 10.1038/s41598-025-05346-w (PMC12217139; doi:10.1038/s41598-025-05346-w)
Supplement: Supplementary file 1 — Supplementary Material 1 [file 41598_2025_5346_MOESM1_ESM.docx]

**Supplementary Information**


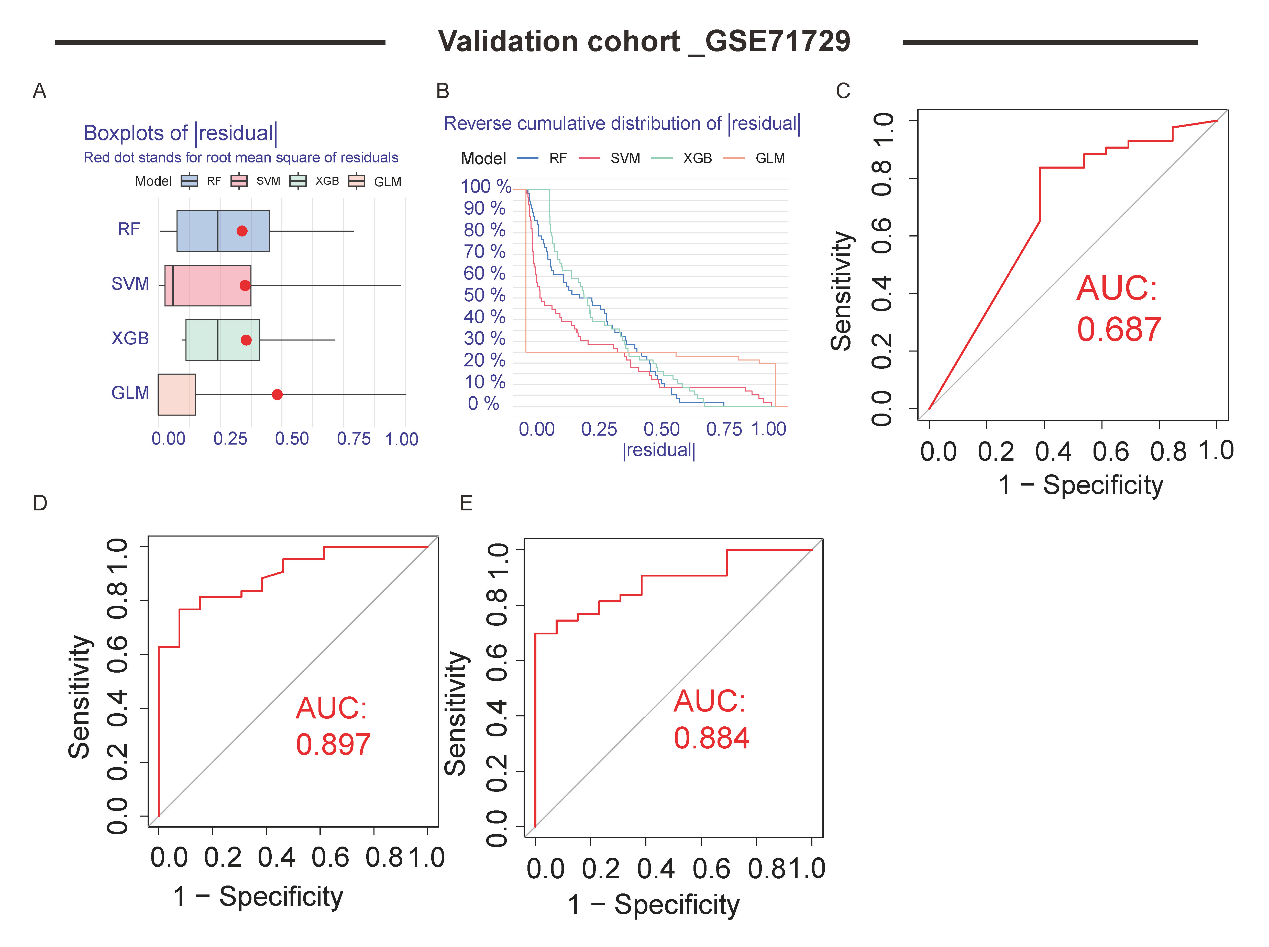


Supplementary Figure 1 (A) Boxplots of residuals for RF, SVM, XGB, and GLM models in the validation cohorts, with lower residuals indicating better performance. (B) Cumulative distribution of residuals for each model, with steeper curves indicating better performance. (B-E) ROC curves in the validation cohort for GLM, RF, and XGB models, respectively

**Supplementary Table 1. 52 key targets of curcumin in pancreatic cancer**

| **Gene name** | | | |
| --- | --- | --- | --- |
| AKT1 | IL2 | CTNNB1 | NFE2L2 |
| TP53 | IL4 | PTGS2 | CCND1 |
| TNF | CASP8 | PPARG | EGR1 |
| IL6 | FOXO1 | TLR4 | MAPK8 |
| STAT3 | MYD88 | MTOR | STAT1 |
| IL1B | HMOX1 | IL10 | NANOG |
| CASP3 | CD34 | CD44 | HSP90AA1 |
| BRCA1 | TGFB1 | IL1A | HIF1A |
| PTK2 | MMP9 | SIRT1 | BCL2 |
| TJP1 | MYC | NFKB1 | AREG |
| PTPN11 | MAPK3 | CASP9 | AXL |
| VIM | IL17A | CDH1 | MET |
| TERT | PDGFRB | SOX9 | MMP2 |

**Supplementary Table 2. Molecular docking of Curcumin binding energy results for five targets.**

| Ligands | Receptors | PDB | Structure Weight | Binding Affinity |
| --- | --- | --- | --- | --- |
| Curcumin | CTNNB1 | 1g3j | 128.99kDa | −9.705kcal/mol |
| Curcumin | HIF1A | 1h2k | 45.23kDa | −9.771kcal/mol |
| Curcumin | AREG | 2rnl | 5.58kDa | −147.879kcal/mol |
| Curcumin | VIM | 3g1e | 8.97kDa | − 8.371kcal/mol |
| Curcumin | CASP9 | 1jxq | 126.79kDa | − 11.496kcal/mol |
